# Flexible IMU Sensor Array For 3D Colonoscope Shape Reconstruction and AI‐Based Loop Detection

**DOI:** 10.1002/advs.75119

**Published:** 2026-04-22

**Authors:** Tuukka Panula, Anni Halkilahti, Andrei Ivanov, Matti Kaisti

**Affiliations:** ^1^ Department of computing University of Turku Turku Finland

**Keywords:** ai, colonoscopy, imu, looping, sensor

## Abstract

Looping during colonoscopy procedures is a major cause of patient discomfort and increases the risk of complications. With the rising incidence of colorectal cancer, there is an urgent need for safer and more efficient colonoscopy techniques. We developed a proof‐of‐concept platform for 3D visualization of the colonoscope combined with artificial intelligence (AI)‐based loop detection. The system consists of an array of 15 inertial measurement units (IMUs) mounted on a flexible printed circuit board that can be retrofitted into the instrument channel of conventional colonoscopes. Using sensor fusion algorithms, the position and orientation of individual IMUs are estimated to reconstruct the 3D shape of the colonoscope in real time. The system successfully reconstructed the colonoscope shape in a silicone colon phantom, and the AI model achieved high loop‐detection performance with an area under the receiver operating characteristic curve of 0.95 on test data. These results demonstrate feasibility in an artificial environment, while further validation in clinical settings is required.

## Graphical abstract

We propose a sensing platform for 3D visualization of the colonoscope combined with artificial intelligence (AI)‐based loop detection. The system consists of an array of 15 inertial measurement units (IMUs) mounted on a flexible printed circuit board that can be retrofitted into the instrument channel of conventional colonoscopes.

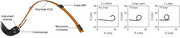

## 1 Introduction

Colorectal cancer (CRC) is the third most common cancer and the second leading cause of cancer mortality worldwide. By 2040, annual incidence rates of CRC are estimated to increase to 3.2 million cases, while mortality is estimated to reach 1.6 million cases [1]. Furthermore, there has been a notable global increase in early‐onset CRC, affecting individuals aged 25 to 50 [2]. In response to the increasing prevalence of CRC, numerous national medical boards have implemented standardized protocols for CRC screening. These protocols aim to detect CRC earlier, thus reducing mortality rates associated with the disease [3].

Colonoscopy is the gold standard method for diagnosing CRC [4]. However, performing the procedure requires a highly trained and experienced colonoscopist. The technical difficulties, anatomical variations, and patient discomfort of the procedure contribute to longer procedures, which is a major limitation in carrying out effective CRC screenings [5] Relying primarily on camera images and the colonoscopist's expertise for navigation, the procedure can often be painful for the patient. Inaccurate movements can cause the colonoscope to advance unnecessarily, resulting in loop formation and consequent pain. Looping is a major cause of pain experienced during colonoscopy and the most common cause of incomplete colonoscopies [4, 6]. Looping occurs when the semi‐rigid colonoscope tubing is forced into certain formations, resulting in stretching the colon walls. The risk of perforation increases when loops are undiagnosed or misdiagnosed. Furthermore, approximately 69% of loops are incorrectly detected [4, 7].

Previous efforts to track the position of the colonoscope include various methods. The use of bending sensors embedded in the colonoscope has been used to detect looping [8]. A recent study explored the use of optical fiber Bragg grating sensors in 3D visualization of the colonoscope [9]. The ScopeGuide system, developed by Olympus Corporation, uses coils placed throughout the colonoscope and an external electromagnetic radar unit that probes the position of the colonoscope [10]. This system is in widespread clinical use and has shown potential in the training of colonoscopists [11]. The main application fields in the use of artificial intelligence (AI) in colonoscopy are video feedback‐based lesion detection and autonomous colonoscope maneuvering [12, 13, 14, 15]. Recent efforts have focused on increasing the accuracy of electromagnetic 3D tracking using AI [16, 17]. With the increase in CRC incidences and mortality, there is an urgent need for safer and more efficient colonoscopy procedures. However, these methods require the use of external hardware or new equipment. This leads to high acquisition costs, which prevent the widespread clinical adoption of these technologies.

The development of micro‐electromechanical systems (MEMS) has enabled the development of small form factor inertial measurement units (IMU), small enough to fit into the instrument channel of an colonoscope. An IMU comprises a three‐axis accelerometer and a three‐axis gyroscope, embedded in a single package. The majority of the research on the use of IMUs in colonoscopy has been limited to tracking an ingestible capsule colonoscope [18, 19]. The use of IMUs attached to a flexible tubing has been proposed [20]. While promising, the approach has not yet been sufficiently miniaturized for practical integration into clinical colonoscopes. In our previous study, we showed the possibility of tracking the tip of the colonoscope using a single IMU while advancing the instrument in constant increments [21].

**FIGURE 1:**
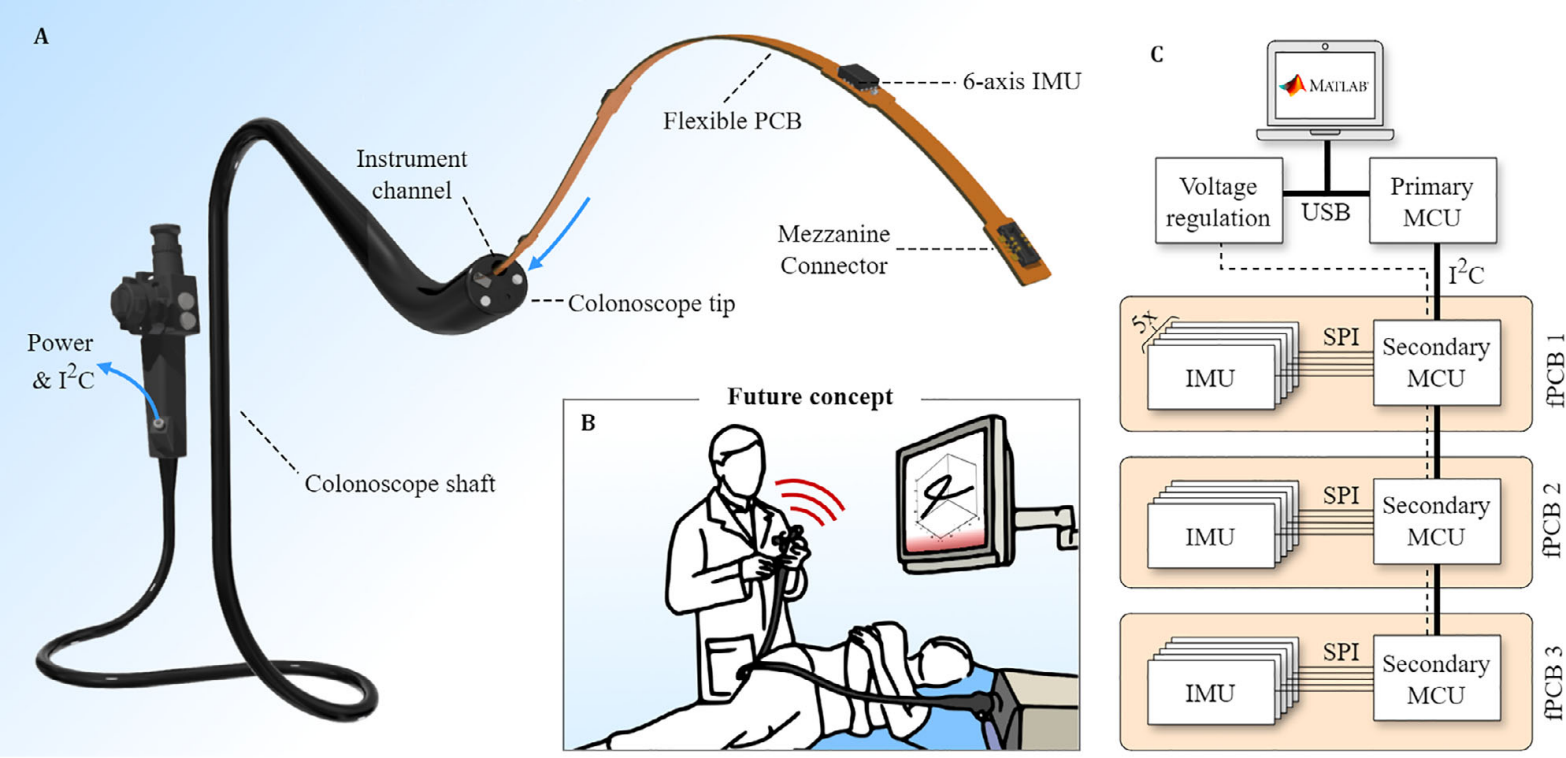
System concept. (a) The fPCB array consisting of 15 IMUs is inserted into the instrument channel of the colonoscope. (b) Future development includes the implementation of real‐time feedback to the colonoscopist in case of looping. Real‐time operation is not implemented in this study. (c) System diagram of the proposed setup. Three fPCBs, each housing five IMUs and an MCU, are inserted into the colonoscope's instrument channel. The primary MCU acts as an interface between the sensing array and a computer.

We have developed a system to reconstruct the shape and orientation of the colonoscope using IMUs, with the future use case depicted in Figure 1b. The system consists of an array of IMUs placed inside the instrument channel of a typical colonoscope routinely used in clinics, as shown in Figure 1a,c. Using sensor fusion algorithms on the collected data, the system enables 3D visualization of the colonoscope. Using data collected with this device, we trained a machine learning model capable of detecting looping in the colon. Our solution achieves 3D tracking without external instrumentation and can be retrofitted to existing colonoscope systems at a very low manufacturing cost. The trained AI model is expected to improve diagnostic accuracy, reduce patient discomfort and colonoscopy failure rates by lowering the risk of looping complications, thus optimizing healthcare resources.

The main contributions of this work are: 1) the development of a retrofit IMU‐based platform for colonoscope shape sensing, 2) real‐time 3D reconstruction of the colonoscope using distributed inertial sensing and sensor fusion as well as 3) an AI‐based approach for automatic loop detection.

## 2 Results

### 2.1 System Description

The system integrates a sensor platform and algorithms for tracking the shape of the colonoscope and loop detection. The sensor platform consists of a primary microcontroller unit (MCU) located outside the colonoscope and a sensing array designed to be integrated within the instrument channel (⊘=3.2mm) through the opening in the instrument control head. The sensing array consists of three flexible printed circuit boards (fPCBs) housing a total of 15 IMUs and three secondary MCUs.

The primary MCU is responsible for interfacing with the computer via a Universal Serial Bus (USB) connection, functioning as the master controller capable of requesting data from the secondary MCUs through the $I^{2}C$ bus.

**FIGURE 2:**
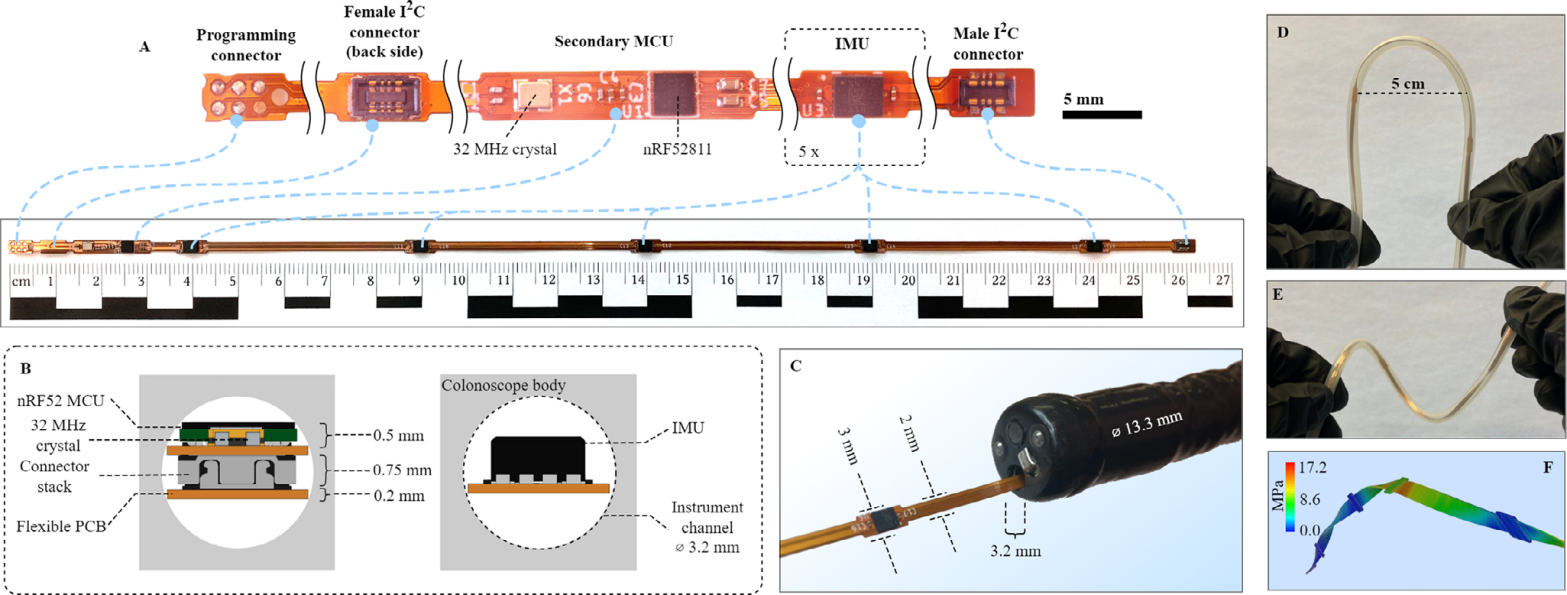
Hardware setup. (a) A microphotograph of a single fPCB with all of the electronic components. (b) Cross‐section view of the fPCB inserted into the instrument channel of the colonoscope. The dimensions are indicative, rather than exact. (c) A photograph of the fPCB being inserted into the distal end of an Olympus colonoscope. (d,e) The fPCB inserted into plastic tubing in order to visualize the bending of the fPCB. (f) FEM analysis of the fPCB with a 90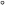 turn between subsequent IMUs.

The sensing array is composed of three fPCBs that are daisy‐chained via mezzanine connectors that provide power and $I^{2}C$ communication. Each fPCB houses a male and a female connector at the opposite ends. The connector stack is shown in Figure 2b. Each fPCB contains one secondary MCU and five IMUs, as shown in Figure 2a, for a total of 15 IMUs distributed along the 70 cm sensing array. Each secondary MCU controls the five IMUs located on its respective fPCB, and these secondary MCUs are connected to a shared $I^{2}C$ bus that extends throughout the series of fPCBs.

A 2 mm diameter four‐wire cabling was soldered to a custom interface PCB and connected to the mezzanine connector at the end of the sensing array. This cabling, featuring power and $I^{2}C$ lines, is connected to the primary MCU outside the colonoscope. The dimensions of the sensing array and its components were selected to be compatible with most commercial colonoscopes, with a maximum assembly width of 3 mm and a maximum component stack thickness of 1.45 mm, as shown in Figure 2b,c.

Since fPCBs are rigid along their transverse axis, we incorporated 90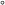 longitudinal twists in the fPCBs of the sensing array before inserting the sensing into the instrument channel. This allows the array to follow the colonoscope's curvature freely and avoids sudden rotation along the longitudinal axis during navigation.

The impact of bending the sensing array is demonstrated in Figures 2d,e where a single fPCB inserted into a 3 mm diameter plastic tubing.It was observed that the 90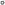 twist applied to the fPCB reduces the erratic longitudinal twisting when a transverse bend occurs. Finite Element Method (FEM) analysis in Figure 2f demonstrates that when bent, the strain in the fPCB is distributed across alternating segments of the fPCB. The material used for simulation was 0.2 mm thick polyimide. The highest strain is located at the 5 cm‐long connecting parts between IMUs, rather than at the IMUs themselves. Since the maximum possible bending radius of the colonoscope is limited to approximately 5 cm, the fPCB will not be forced into bends exceeding its structural limits. Additionally, the 5 cm distance is small enough to be able to capture even the tightest looping scenario. The polyimide material used is very flexible, and its effect on the colonoscope handling is very minimal ‐comparable to that of the cable actuated forceps used in the instrument channel. The simulated Von Mises stress from the FEM analysis shows that the highest strain is observed at transition from the connecting part (11.6 MPa) to the IMU section, where it islower (7.9 MPa). The segment housing the ball grid array (BGA) mounted MCU is the most vulnerable to failure; no such failures were detected during extensive testing. After approximately 100 actual measurements, the MCU showed no signs of failure.

To address all 15 IMUs, the primary MCU communicates via unique $I^{2}C$ addresses with three fPCBs, each housing a secondary MCU. On each respective fPCB, the secondary MCU accesses the five local IMUs using SPI and individual chip select (CS) lines.

The secondary MCU collects IMU data from all five units at a frequency of 15 Hz and transmits the data through $I^{2}C$ when requested by the primary MCU. The primary MCU timestamps each data transfer, consisting of data from five IMUs, with the $I^{2}C$ address of the corresponding secondary MCU. The data is outputted as 16‐bit hexadecimal values at a rate of 15 Hz over a USB connection and data is saved on PC for offline analysis. Real‐time data visualization was not implemented in this setup. Each secondary MCU is programmed via a 6‐pin programming connector (Segger J‐Link, Germany). We analyzed the $I^{2}C$ bus during the measurement using an oscilloscope. Transmission time for a single secondary MCU, consisting data from 5 IMUs was 6 ms. Detailed analysis of the $I^{2}C$ transfer can be found in Supporting Information.

Following the recording phase, the IMU data, initially represented in two's complement format, is converted into physical units with three axes of acceleration a (m $s^{-2}$) and the three axes of angular velocity ω (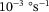). Data acquisition produces a 15×6 matrix per sample, which we call a frame. With a sampling rate of 15 Hz, the system generates a total of 1,350 data points per second. Subsequently, a six‐axis Kalman filter K is applied to each individual IMU recording.

(1) $$q_{i}=K\;\left(a_{i},\omega_{i},\theta_{i}\right),$$

where i=1,⋯,15 is the IMU index, θ are the filter tuning parameters and $a,\omega \in R^{3}$. The Kalman filter processes the X, Y, and Z values from both the accelerometer and gyroscope as inputs, along with the IMU specific filter parameters. The filter outputs the orientation of a single IMU in quaternion representation.

### 2.2 Electronics

Custom polyimide (PI) fPCBs were manufactured to house the individual sensors. The fPCB houses an array of five LSM6DSO (Bosch Sensortec, Germany) IMU sensors placed 50 mm apart, an nRF52811 (Nordic Semiconductor, Norway) wafer‐level chip scale packaged MCU, 32 MHz crystal oscillator (Raltron, USA), and two board‐to‐board connectors (Molex, USA). The 0.2 mm thick fPCB is made from flexible polyimide plastic with four signal layers and has a maximum width of 3 mm. Only through‐hole vias were used. Copper traces were made on an 18 μm thickness and an immersion gold (ENIG) surface finishing process. A minimum width of 0.15 mm was used for the signal traces.

In addition, a small two‐layer PI fPCB, with a board‐to‐board connector and four solder pads, was designed in order to connect the sensing array to a four‐wire cable assembly. The wires are routed to the primary nRF52840 (Nordic Semiconductor) MCU. The sensing array is powered using an adjustable LM317T (Texas Instruments, USA) linear regulator set at 3 V.

### 2.3 Sensor Characterization

The accelerometer was configured with a measurement range of ±2g and the gyroscope with a range of ±125dps. The sensor provides 16‐bit resolution, corresponding to approximately 0.061mg/LSB for the accelerometer and 4.375mdps/LSB for the gyroscope. The accelerometer exhibits a typical noise density of approximately $60\;\mu g/\sqrt{\mathrm{Hz}}$, while the gyroscope noise density is approximately $4\;\mathrm{mdps}/\sqrt{\mathrm{Hz}}$.

**FIGURE 3:**
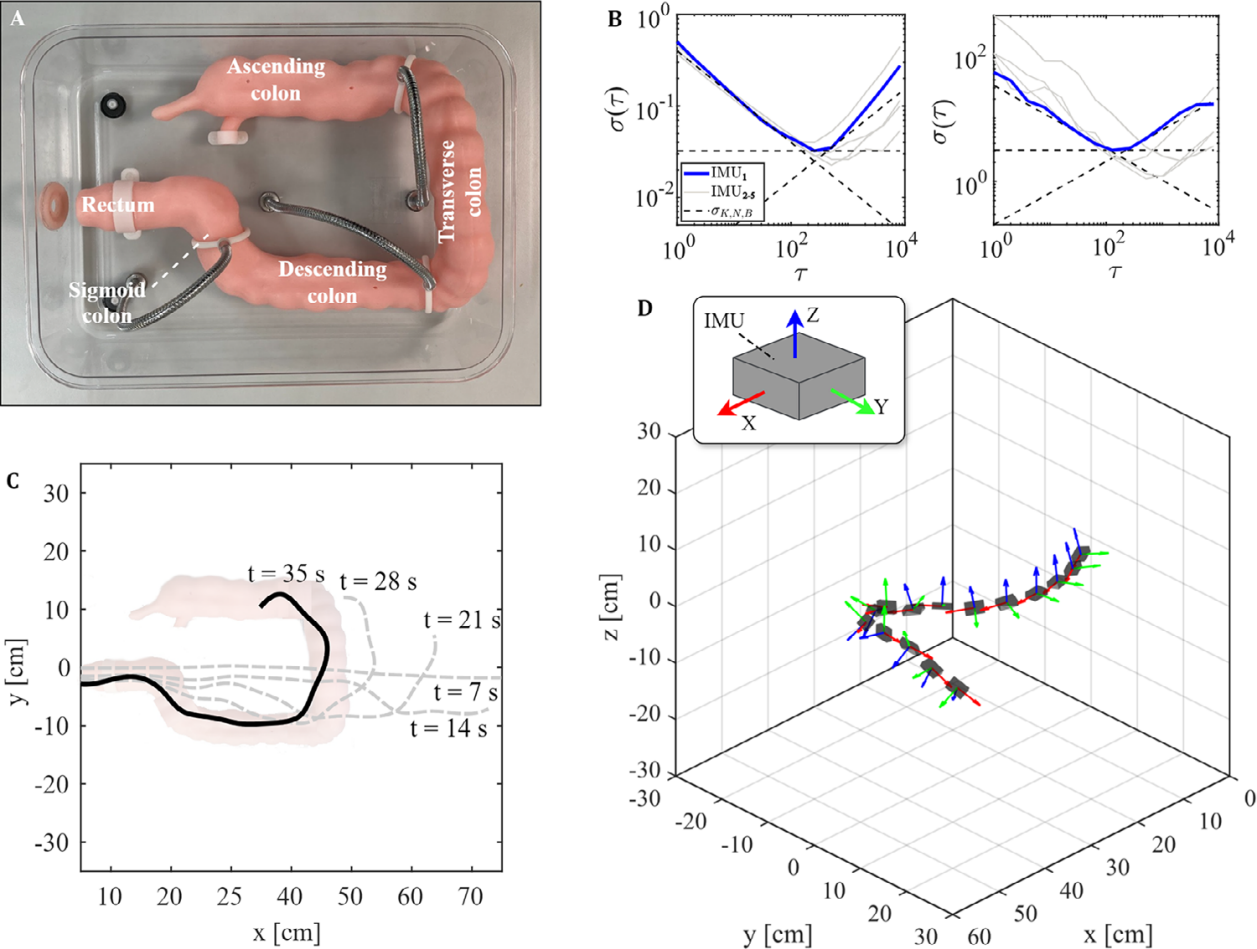
Experimental results. (a) A photograph of the silicone colon phantom. (b) Example Allan deviation plots from a single fPCB, with accelerometer X‐axis on the left and gyroscope X‐axis on the right. The dashed lines show the computation of N (angle random walk), K (rate random walk), and B (bias instability). (c) A measurement taken using the silicone colon phantom. 7 s time steps are shown to visualize the movement of the colonoscope. d) A 3D plot showing a single frame with the position and orientation of each IMU inside the colon phantom.

Although all 15 IMUs are of the same type, each unit must be analyzed individually due to variations in their noise characteristics. The assembled sensor array was placed on a table, and the gyroscope and accelerometer outputs were recorded for one hour at a fixed sampling rate. We were able to assess the characteristics of each IMU separately. The results from Allan deviation analysis indicate that manufacturing tolerances influence both gyroscope and accelerometer characteristics, with the effect being particularly pronounced in the gyroscope. Two example Allan deviation plots are shown in Figure 3c, where the single‐axis results from one fPCB with five IMUs are compared.

The accumulation of orientation error was analyzed by taking a static 20 min measurement and assessing the drifting of the angle between each consecutive IMUs. A drift rate of 2.1 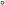 min^−1^ was observed.

### 2.4 3D Tracking Algorithm

The entire recording consists of the fPCB addresses along with all the axes of accelerometer and gyroscope data from each of the five IMUs on the respective fPCB. This recording is then preprocessed to remove linear drift associated with gyroscope data. The Kalman filter, which is used to fuse accelerometer and gyroscope data to estimate its orientation, is then applied for each IMU separately. The filter is tuned with parameters derived from Allan deviation analysis conducted on a static measurement. The detailed parameter extraction can be found in the Methods section. This process is used to compute an array of 15 quaternions, each representing the orientation of an IMU. At the beginning of each measurement, the colonoscope is positioned in a straight starting configuration on the table, which serves as a basis for calibrating the quaternions to a common reference orientation.

Starting from the first IMU (proximal end), matrix rotations are performed for each IMU separately. Orientation at the IMU is defined by a quaternion q and normalized as $q_{n}$:

(2) $$q=a+b\;i+c\;j+d\;k,\;q_{n}=\frac{q}{\sqrt{a^{2}+b^{2}+c^{2}+d^{2}}}.$$

where i, j, and k are the base vectors and a, b, c and d are real numbers. We define the vector u that points forward (along the sensor's x‐axis). This vector is then converted into its pure‐quaternion representation $u_{q}$:

(3) $$\begin{array}{cccc} u & =\left[\begin{array}{c} 5\\ 0\\ 0 \end{array}\right], & u_{q} & =\left[\begin{array}{c} 0\\ u \end{array}\right]=0+5i+0j+0k. \end{array}$$

u is then rotated in the Cartesian coordinate system using the normalized quaternion $q_{n}$:

(4) $$s_{q}=q_{n}\;u_{q}\;q_{n}^{*},\;q_{n}^{*}=a-b\;i-c\;j-d\;k.$$

where $q_{n}^{*}$ is the complex conjugate of $q_{n}$. $s_{q}$ is finally transformed back to its vector representation $s_{i}={\left[x\;y\;z\right]}^{T}$, where i depicts the order of the IMU starting from the proximal end. The process is repeated for each of the 15 IMUs, appending the vector array by 5 cm each time, resulting in:

(5) $$S=\left[\begin{array}{c} s_{1}^{⊤}\\ s_{2}^{⊤}\\ \vdots \\ s_{15}^{⊤} \end{array}\right]\in R^{15\times 3}.$$

S depicts a single frame, which consists of the 3D locations of all IMUs, giving a snapshot of the colonoscope's position, representing the full IMU array in Cartesian coordinates in cm. Rather than tracking each IMU as an independent point in space, we recover the colonoscope geometry from the IMU orientations and the known kinematic linkage: successive IMUs are separated by 5 cm along their local x‐axis, and the global position of each IMU is obtained by chaining the oriented 5 cm link vectors.

We first assessed a simple scenario, with the distal tip of the colonoscope fixed to a vice. Using the control head, the tip of the colonoscope was steered to four directions: up, down, left, and right. The algorithm was able to replicate the steering procedures at the tip of the colonoscope, as shown in Figure 3a.

**FIGURE 4:**
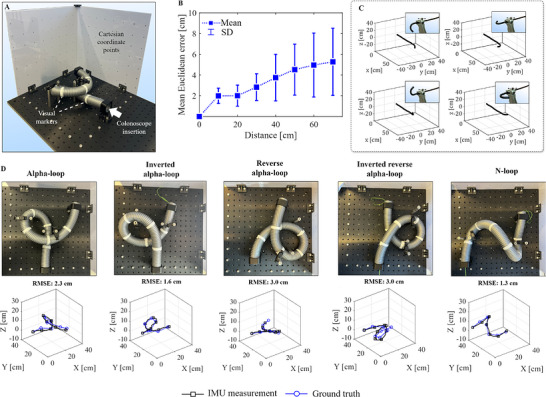
System performance evaluation. (a) Data collection setup with the plastic tubing arranged in a looping formation. (b) Mean Euclidean error propagation can be seen in the distal sensors. (c) The colonoscope tip was manipulated in four directions. (d) Each loop formation was mapped in the 3D coordinate system in order to get the ground truth point cloud. The markers on the plastic tubing are placed 10 cm apart. The bottom figures show measurements taken with the sensing system plotted against the ground truth positions.

In order to quantify the performance of the proposed system, we analyzed 60 measurements taken with the sensing array and compared them to ground truth values taken from plastic tubing models (Figure 4a). The analysis was done on a frame corresponding to the moment where the sensing array was fully inserted. The average root“mean”square error (RMSE) over all measurements was 3.79±1.64 cm. The same analysis was run on results computed without individual tuning for the Kalman filter, resulting in 4.1±2.6 cm. It should be noted that the tubing diameter was 4 cm, which introduces an inherent geometric uncertainty in the measured positions. Example measurements from each loop type are depicted in Figure 4d and all loop types are reported in Supporting Information. Figure 4b shows the mean Euclidean error of IMUs placed between 10 cm intervals. The propagation of error along the tube can be observed, with errors increasing at more distal positions.

The ability of the sensing array to perform in a realistic scenario was assessed using a silicone colon phantom, which is shown in Figure 3b. The colonoscope equipped with the developed sensing array was maneuvered through the colon phantom as shown in Figure 3d. The data was collected via USB connection and analyzed only in postprocessing. In Figure 3e, the entire sensing array is depicted in a 3D plot (60 × 60 × 60 cm), with each IMU visualized as a separate object showing its spatial position and orientation. Because all IMUs were calibrated to a common reference orientation at the start of the measurement, the twisting of the fPCBs is not visible in the resulting plot.

### 2.5 Loop Detection Algorithm

To showcase the system's potential to detect looping of the colonoscope, we collected separate training and test datasets and applied two different AI models to the data. All data was initially processed using the 3D tracking algorithm. To construct the input vector for the AI model, the 3D positions of all IMUs were concatenated into a single feature vector X and appended with the corresponding label.

(6) $$X=\left[\begin{array}{c} s_{1}\\ s_{2}\\ \vdots \\ s_{15} \end{array}\right],\;s_{i}=\left[\begin{array}{c} x_{i}\\ y_{i}\\ z_{i} \end{array}\right]\in R^{3}.$$

Next, each frame was subjected to a random rotation in order to augment the dataset. The rotation changes the overall orientation of the colonoscope, so the relative distances between individual IMUs stay the same.

(7) $$\tilde{X}=\left[\begin{array}{c} R\left(\theta \right)\;s_{1}\\ R\left(\theta \right)\;s_{2}\\ \vdots \\ R\left(\theta \right)\;s_{15} \end{array}\right],$$

where $R\left(\theta \right)=R_{x}\left(\theta_{x}\right)\;R_{y}\left(\theta_{y}\right)\;R_{z}\left(\theta_{z}\right)$ is the rotation matrix and $\theta ={\left[\theta_{x},\theta_{y},\theta_{z}\right]}^{⊤}$ represents the rotation angles about the x, y, and z axes, and $\theta_{x}\;\theta_{y},\theta_{z}\in \left[-20^{\circ },20^{\circ }\right]$ These rotation angles have been chosen to reflect the range of rotations possible within the physical constraints of the colonoscope, as well as making sure the rotations do not change the loop type.

**FIGURE 5:**
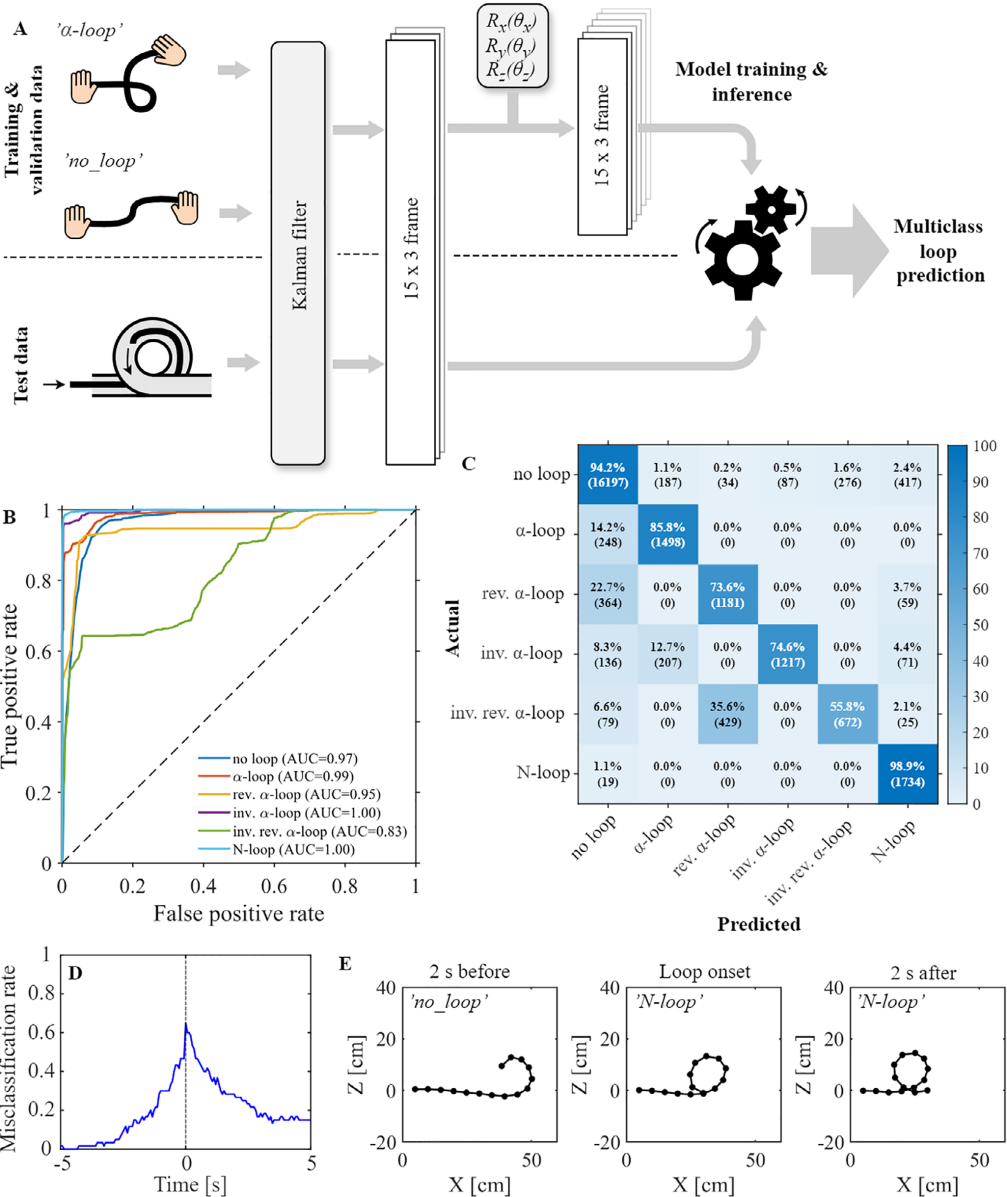
AI‐based loop detection. (a) Pipeline of the data preparation for both training and testing. (b) Confusion matrix of the result of the logistic regression model using the test data set. Each value represents a single frame with 15 IMUs. (c) Receiver operating characteristic curves of each class. Macro‐AUC was 0.95. (d) Misclassification rate increases near the onset of looping. (e) Labeled frames from the loop detection model. The left plot is labeled as no loop, 3 s before the onset of looping. The middle and the right plots are labeled as a loop, with the onset in the middle.

Two AI models were applied: logistic regression and a support vector machine (SVM) for multi‐class loop classification. These include the following five classes: no looping, α‐loop, reverse α‐loop, inverted α‐loop, inverted reverse α‐loop and N‐loop [22]. Five‐fold cross‐validation was used to validate the models using only the training data set. The cross‐validation accuracy was high for both models: 98.97% and 99.62% for logistic regression and SVM, respectively. Using the trained models on the test data, the macro area under the receiving operating characteristic curve (Macro‐AUC) was 0.95 for both logistic regression and SVM, as shown in Figure 5c. Confusion matrix for the logistic regression model results is shown in Figure 5b. The results, using the test data, prove that both models were able to successfully differentiate between looped and non‐looped colonoscope configurations. Furthermore, the model was able to distiguish between different loop types. The inference time for a single prediction (averaged over 1000 samples) was 1.9 ms, making the model well‐suited for real‐time use. Figure 5d illustrates the AI model operating before, during, and after the onset of looping.

## 3 Conclusion

The increasing demand for colonoscopies due to CRC screening programs worldwide highlights the urgent need for safer and more efficient technologies. To address this, we have developed an inertial measurement unit (IMU)‐based sensor platform that allows 3D visualization of the colonoscope. We were able to implement a flexible sensor array compact enough into integrate to a commercial colonoscope. The six‐axis IMU data was fed through a sensor fusion algorithm to reconstruct the shape of the colonoscope. The feasibility of the system was verified using a silicone colon phantom. In addition, we showed the possibility to detect looping in a simulated environment using two separate AI models. The model achieved an AUC of 0.95, suggesting that it is possible to detect looping of the colonoscope with the proposed system.

Despite promising results, there are limitations to the study. This study is an indicative proof‐of‐concept study and the results cannot directly be applied to real‐life clinical use. The silicone model used in the study cannot reproduce looping, and within the scope of this study, our looping data collection focused solely on five loop types that can form in the sigmoid colon. The use of a realistic, clinical‐training colonoscopy phantom would mitigate this limitation by providing anatomically accurate compliance and looping behavior. Although our training data for the loop detection was large, it had only 30 independent loop formations, which were then augmented to five different artificial rotations. The collection of more independent measurements on a realistic colon phantom that can reproduce looping, would be needed for preclinical validation. The training data were manually labeled and therefore, may not always accurately reflect the true looping state of the colonoscope. In future work, consultation with experienced colonoscopist for data annotation and validation would help mitigate this limitation and improve label reliability. In our results, there was a relatively large misclassification of an inverted reverse α loop as reverse α loop. Similar phenomenon was observed to a lesser extent between α loops and inverted α loops. This is likely due to their shapes being very close to another. In future work, minimizing the classification of one loop type as another is crucial, as such errors can cause an increased perforation risk. The use of clinical‐training phantom as well as improving label reliability would help mitigate this issue.

Sensor drift is a well‐documented issue in IMU‐based systems. The perceived drift rate was fairly high at 2.1 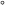 min^−1^. As seen in our results, the error propagates along the kinematic chain. Mitigation techniques include zero‐velocity updates and the use of kinematic constraints. This information can be used to reset drift and update bias estimates. In this study, we use a simple Kalman filter, and each IMU is considered as a separate entity only linked by the 5 cm kinematic linkage. Switching from commmercial grade to industrial grade IMU and the application of more sophisticated algorithms could potentially reduce the error significantly [23].

Compared to the clinical gold‐standard electromagnetic tracking method, which has reported errors of approximately 12.4 mm, the accuracy of the proposed approach remains lower [24].

Future developments include real‐time 3D tracking, the implementation of a user interface, along with a feedback system to prevent looping. Although the proof‐of‐concept AI‐based loop detection showed great promise, further exploration is needed. Predictive loop detection would enable the colonoscopist to apply steering measures before the loop occurs, significantly increasing patient comfort. In addition, in the current study, we did not record video. The video feed has been used to estimate the position of the colonoscope and to reconstruct the trajectory of the instrument [25]. A promising sensor fusion approach could be using the optical flow data gained from the video feed and the developed sensing array.

The developed system has potential applications in other fields of medicine as well. Surgical tracking systems are used especially in minimally invasive procedures [26]. Current systems include optical and electromagnetic solutions [27, 28]. Possible use cases include catheter guiding, laparoscopy, and radiotherapy.

There is still a long way of translating laboratory data into actual clinical applicability. The current looping models are rigid and highly simplified – unable to capture the different anatomies between people as well as the dynamic nature of the colonoscopy procedure. Furthermore, our data does not account for the event of two simultaneous loops. Even before human studies, the technology has to be validated in a simulated colonoscopy training environment. Being an invasive procedure, in order for us to be able to conduct human studies, electrical safety, the electromagnetic compatibility and overall usability has to be rigorously validated. The usability is also highly dependent on the system's capability to provide reliable data for the whole duration of the procedure. Moving forward, multi‐center data collection to improve the AI model generalization is crucial. However, this study represents an initial proof‐of‐concept with promising results.

## 4 Experimental Section

### 4.1 Software

Firmware for the primary and secondary MCUs was written in C using Segger Embedded Studio 7.30 integrated development environment (IDE). The electronics were designed using Autodesk Eagle 9.6.2 electronic design automation (EDA) software. FEM was done on Autodesk Nastran In‐CAD software. The script for data acquisition via USB serial connection was implemented on Python 3.12. Processing of the IMU data was performed on MATLAB R2024a using the following toolboxes: Sensor Fusion and Tracking, Navigation, Signal Processing, as well as Statistics and Machine Learning. AI model training and inference were performed on a workstation equipped with an Intel Core Ultra 7 165H central processing unit (16 cores, 22 threads, 3.8 GHz base frequency) and 32 GB of RAM.

### 4.2 Noise Parameter Estimation

The frequency stability of each sensor was assessed with Allan deviation analysis. Allan deviation σ(τ) was computed using:

(8) $$\sigma_{y}\left(\tau \right)=\sqrt{\frac{1}{2\left(N-2\right)\tau^{2}}\sum_{n=1}^{N-2}{\left({\bar{y}}_{n+2}-2{\bar{y}}_{n+1}+{\bar{y}}_{n}\right)}^{2}}$$

where ${\bar{y}}_{n}$ is the average of the measured signal over the n‐th sampling interval of duration τ, and N is the total number of samples.

The standard inertial sensor noise coefficients N (angle / velocity random walk), K (rate random walk) and B (bias instability) were computed from σ(τ) as:

(9) $$N=\sigma \left(\tau \right)\sqrt{\tau },\;\text{(slope = -0.5)}$$

(10) $$K=\sigma \left(\tau \right)\sqrt{\frac{3}{\tau }},\;\text{(slope = +0.5)}$$

(11) $$B=\frac{\sigma_{\min}}{0.664}$$

The units for N, K, and B are $m/s^{2}/\sqrt{\mathrm{Hz}}$, $m/s^{2}\sqrt{\mathrm{Hz}}$, and $m/s^{2}$ for the accelerometer, and 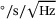, 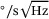, and 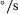 for the gyroscope, respectively. N quantifies the effect of white noise in the sensor output. K characterizes low‐frequency noise that causes the sensor's bias to drift as a random process. B represents the flicker or pink noise contribution. The noise parameters were used to individually tune the Kalman filter for each IMU.

### 4.3 Error Metrics

To evaluate the accuracy of the reconstructed sensor positions, the estimated IMU coordinates were compared with the corresponding ground‐truth colon coordinates at the matched sensor locations. A total of eight matched points were used for each measurement.

First, the point‐wise Euclidean error was calculated for each point as the 3D distance between the estimated position and the ground‐truth position

(12) $$e_{i}={\left\|p_{i}^{\mathrm{GT}}-p_{i}^{\mathrm{est}}\right\|}_{2},$$

where $p_{i}^{\mathrm{GT}}\in R^{3}$ denotes the ground‐truth position of point i obtained from the reference colon model, and $p_{i}^{\mathrm{est}}\in R^{3}$ denotes the estimated IMU‐based position after alignment.

For each measurement, the overall reconstruction accuracy was summarized using the root‐mean‐square error (RMSE) across the matched points

(13) $$\mathrm{RMSE}=\sqrt{\frac{1}{N}\sum_{i=1}^{N}{∥p_{i}^{\mathrm{GT}}-p_{i}^{\mathrm{est}}∥}_{2}^{2}}.$$

In addition, point‐wise Euclidean errors were aggregated across measurements to evaluate spatial error propagation along the colon. For each point index, the mean and standard deviation of the Euclidean error were calculated across the measurement set.

### 4.4 Experimental Setup

An Olympus CF‐100HL colonoscope (Olympus Optical Co., Japan) was used for the experimental setup. The colonoscope features an insertion tube outer diameter of 13.3 and a 3.2 mm instrument channel. The distal end allows bidirectional tip angulation, enabling navigation through the colon. We used a silicone colon phantom (YUYUE YY‐MK1, China), shown in Figure 3b, to verify the operation of the colonoscope equipped with our sensing array. In addition to the silicone model, different loop formations were implemented on a separate setup using flexible plastic tubing. A 70 cm long tubing was marked at 10 cm intervals and fixed to an aluminum breadboard (MB4560/M, Thorlabs, USA) using 3D printed holders. Two marked polycarbonate plates were assembled at 90 degrees in relation to each other and the breadboard in order to be able to visually map the spatial markers on the tubing to the cartesian coordinate system. The setup is shown in Figure 4a.

### 4.5 Data Collection for AI‐Based Loop Detection

Data collection was divided into two parts: training of the loop detection AI models and test data for the AI models. The pipeline of the data preparation for both training and testing is shown in Figure 5a.

Data for training the model were collected by manually forming 30 independent loops consisting of five loop types. Each measurement began with the colonoscope in a straight position, which was then manipulated by hand to create a loop (n = 30). A custom graphical labeling tool was used to identify the approximate frame, where the colonoscope was considered to be in a loop, and label each frame as ‘no_loop’, ‘α_loop’, ‘reverse_α_loop’, ‘inverted_α_loop’, ‘inverted_reverse_α_loop’, or ‘n_loop’. Benefiting from the fact that all values before the onset of looping are labeled as ‘no_loop’ and vice versa, we were able to label a large data set with minimal manual effort. The augmented training dataset consisted of 119700 frames, 63% labeled as ‘no_loop’.

The test dataset was collected by inserting the colonoscope into plastic tubing set to 20 distinct loops. These formations consisted of five different loop types, with three measurements taken from each geometric variation, resulting in 60 different loop measurements. In addition, the test dataset included three measurements taken with the colon phantom. The same labeling tool as for the training part was used to label the onset of looping. The test dataset consisted of 25137 frames, with 68% labeled as ‘no_loop’.

In addition, five measurements were taken using the silicone colon phantom.

## Author Contributions

Conception and design: T.P. and M.K. System development: T.P. and A.I. Collection and assembly of data: A.H. Analysis and interpretation of the data: T.P., A.H. Drafting of the article: T.P., A.H., and M.K., All authors read and approved the final manuscript.

## Conflicts of Interest

The authors declare no conflicts of interest.

## Supporting information


**Supporting File**: advs75119 sup 0001 SuppMat.pdf.

## Data Availability

The data collected and analyzed during the current study are available from the corresponding author on reasonable request.
