# Supplementary material for: Flexible IMU Sensor Array For 3D Colonoscope Shape Reconstruction and AI‐Based Loop Detection
Source: Adv Sci (Weinh). 2026 Apr 22;13(34):e75119. doi: 10.1002/advs.75119 (PMC13285112; doi:10.1002/advs.75119)
Supplement: Supplementary file 1 — Supporting File: advs75119 sup 0001 SuppMat.pdf. [file ADVS-13-e75119-s001.pdf]

Supporting Information

Flexible IMU Sensor Array for 3D Colonoscope Shape Reconstruction and AI-Based Loop Detection

Tuukka Panula, Anni Halkilahti, Andrei Ivanov and Matti Kaisti

Department of computing, University of Turku, Turku, Finland

Data bus analysis

We used a PicoScope 5442D digital oscilloscope along with its accompanying software (Pico Technology, UK) to measure the data transfer in the I^{2}C bus. Each secondary MCU sends the 7-bit address (0x11, 0x12 or 0x13) followed by the IMU data in hexadecimal format. One transfer takes 6 ms. The time between transfers is 22 ms for each secondary MCU. This leaves 16 ms of idle time between transfers in 15 Hz of sampling frequency for all 15 IMUs.

The SDA and SCL waveform quality is adequate at 100 kHz of clock speed. The long lines do exhibit parasitic capacitance that causes spikes of approximately 0.2 V in the SDA line. No dropping packets were observed.

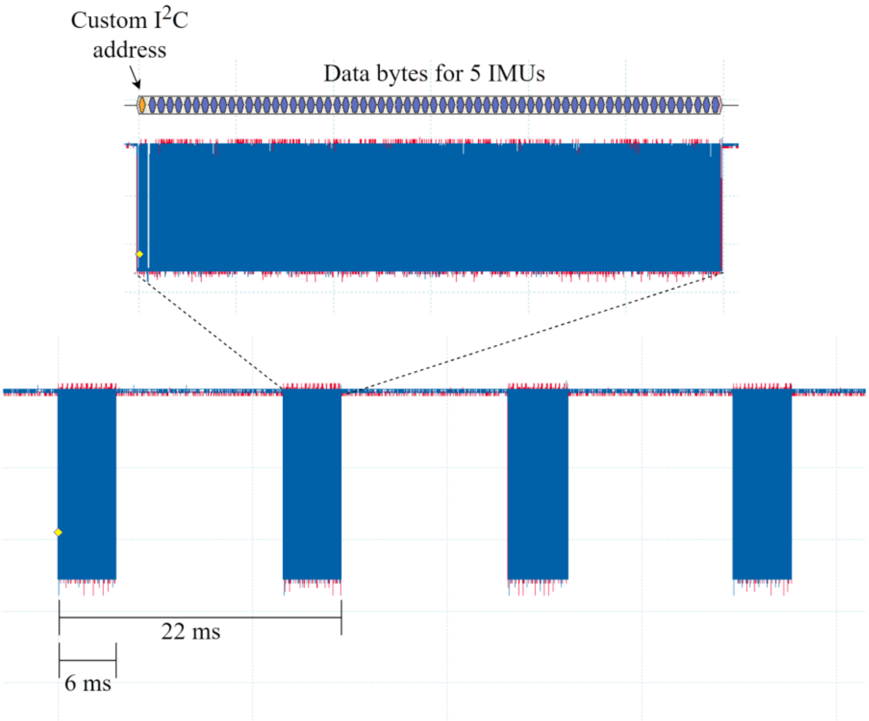

Supplementary Figure 1. Single 6 ms burst contains the data for a single secondary MCU with 5 IMUs. It takes 66 ms for all three secondary MCUs to sample and send the data of 15 IMUs.

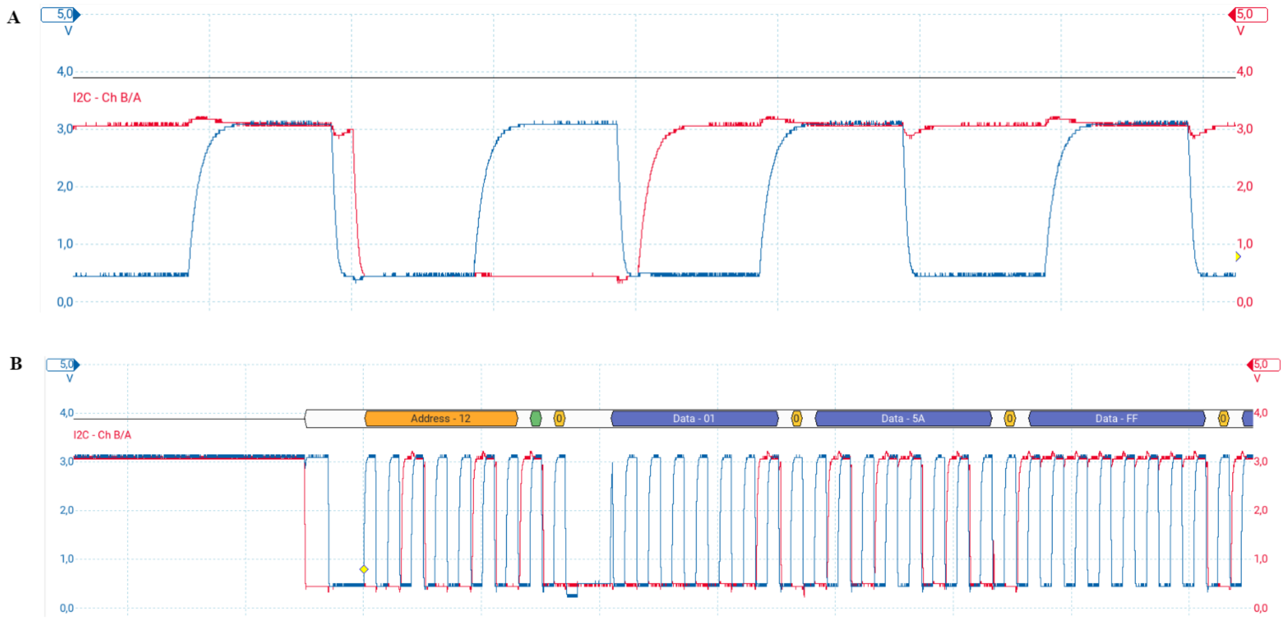

Supplementary Figure 2. a) Closeup screenshot of the SDA (red) and SCL (blue) lines in the I^2C bus. b) Screenshot of an I^2C transfer starting, with the address byte of a single secondary MCU, followed by data bytes.

Loop formations

Supplementary figures 1 to 3 show the 20 different loop formations. Three measurements were taken from each, resulting in a total of 60 measurements.

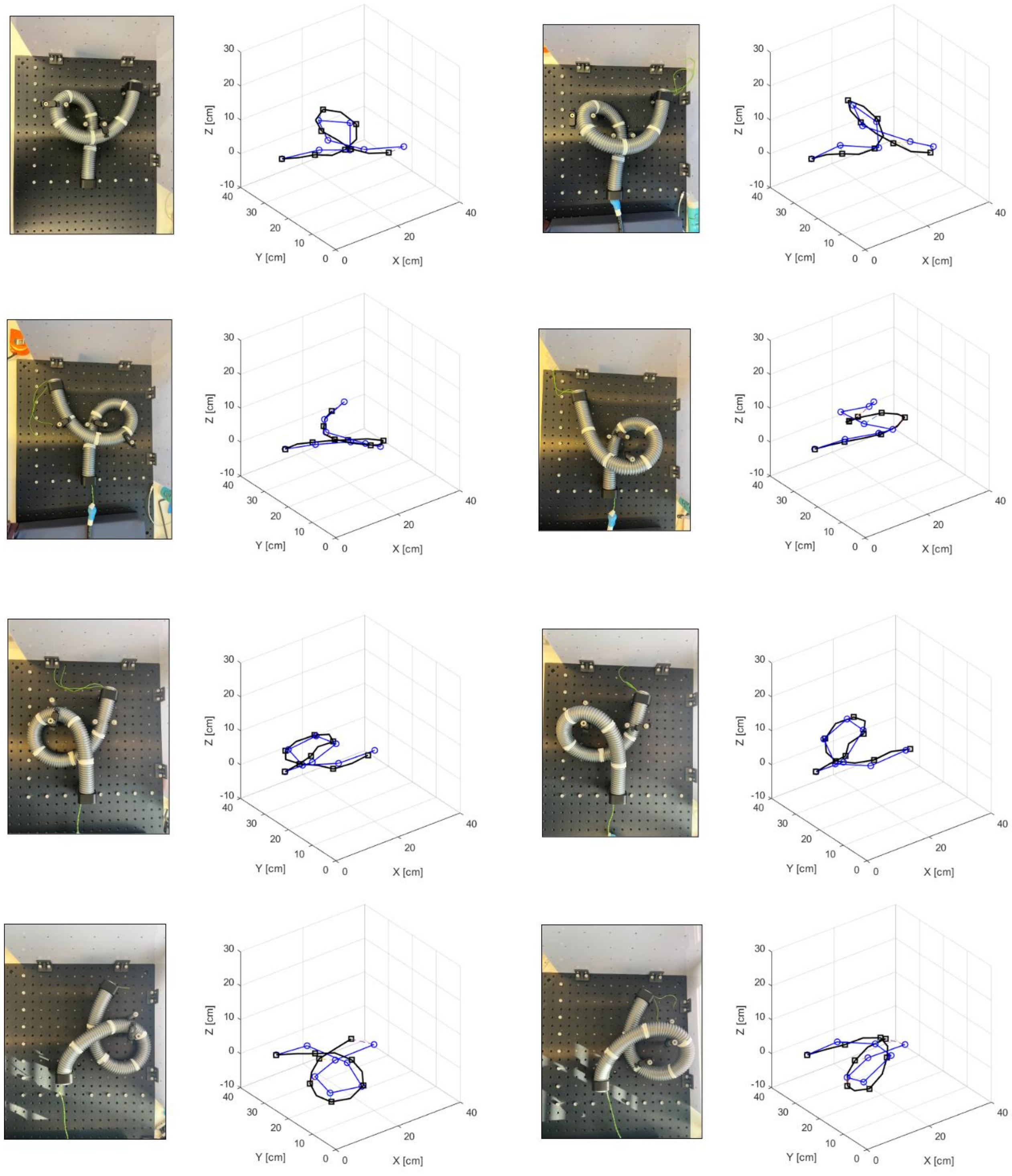

Supplementary Figure 3. 1st row: alpha-loop, 2nd row: reverse alpha-loop, 3rd row: inverted alpha-loop, 4th row: inverted reverse alpha-loop.

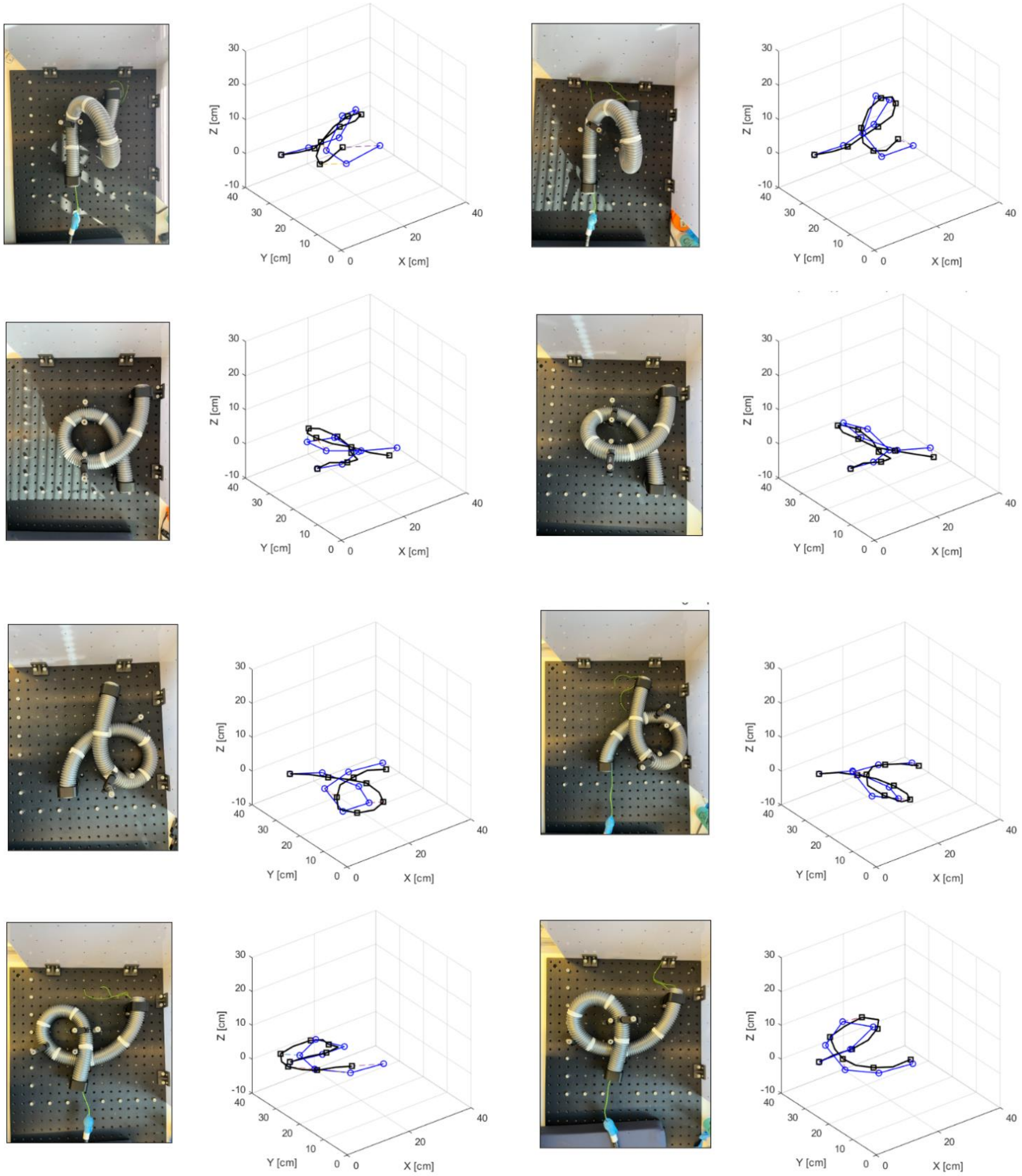

Supplementary Figure 4. 1st row: N-loop, 2nd row: alpha-loop, 3rd row: reverse alpha-loop, 4th row: inverted alpha-loop.

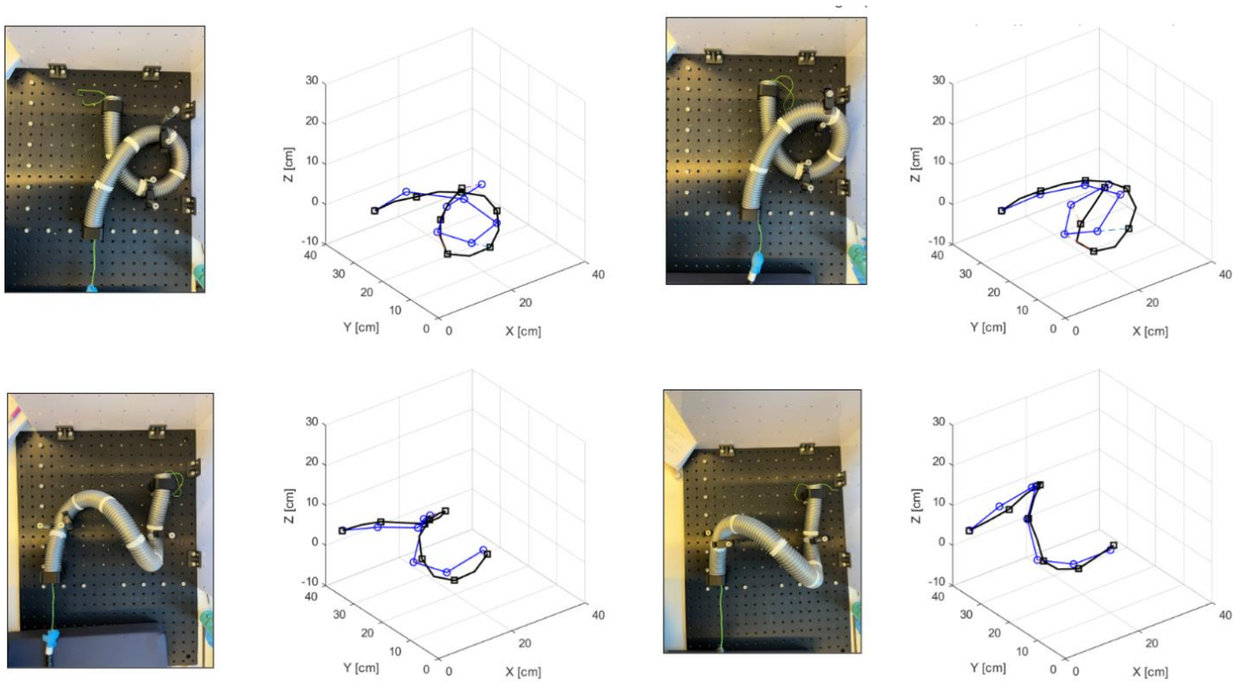

Supplementary Figure 5. 1st row: inverted reverse alpha-loop, 2nd row: N-loop.

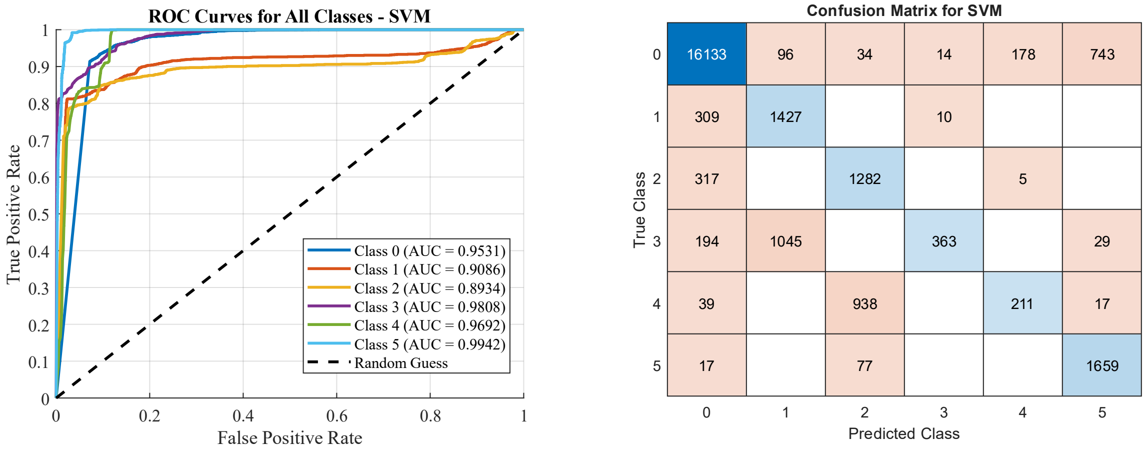

Supplementary Figure 6. ROC curves and confusion matrix for the SVM model. 0: no loop, 1: alpha loop, 2: reverse alpha loop, 3: inverted alpha loop, 4: inverted reverse alpha loop, 5: N loop
